# Mechanical Thrombectomy With and Without Intravenous Tissue Plasminogen Activator for Acute Ischemic Stroke: A Systematic Review and Meta-Analysis Using Nested Knowledge

**DOI:** 10.3389/fneur.2021.759759

**Published:** 2021-12-17

**Authors:** Gautam Adusumilli, John M. Pederson, Nicole Hardy, Kevin M. Kallmes, Kristen Hutchison, Hassan Kobeissi, Daniel M. Heiferman, Jeremy J. Heit

**Affiliations:** ^1^Department of Radiology and Neurosurgery, Stanford University, Stanford, CA, United States; ^2^Nested Knowledge, Inc, St. Paul, MN, United States; ^3^Superior Medical Experts, Inc, St. Paul, MN, United States; ^4^Central Michigan University College of Medicine, Mt. Pleasant, MI, United States; ^5^Semmes-Murphey Clinic, Department of Neurosurgery, Memphis, TN, United States

**Keywords:** stroke, thrombectomy, tissue plasminogen activator, thrombolysis, meta-analysis, semi-automated analysis

## Abstract

**Background:** Mechanical thrombectomy (MT) is now the standard-of-care treatment for acute ischemic stroke (AIS) of the anterior circulation and may be performed irrespective of intravenous tissue plasminogen activator (IV-tPA) eligibility prior to the procedure. This study aims to understand better if tPA leads to higher rates of reperfusion and improves functional outcomes in AIS patients after MT and to simultaneously evaluate the functionality and efficiency of a novel semi-automated systematic review platform.

**Methods:** The Nested Knowledge AutoLit semi-automated systematic review platform was utilized to identify randomized control trials published between 2010 and 2021 reporting the use of mechanical thrombectomy and IV-tPA (MT+tPA) vs. MT alone for AIS treatment. The primary outcome was the rate of successful recanalization, defined as thrombolysis in cerebral infarction (TICI) scores ≥2b. Secondary outcomes included 90-day modified Rankin Scale (mRS) 0–2, 90-day mortality, distal embolization to new territory, and symptomatic intracranial hemorrhage (sICH). A separate random effects model was fit for each outcome measure.

**Results:** We subjectively found Nested Knowledge to be highly streamlined and effective at sourcing the correct literature. Four studies with 1,633 patients, 816 in the MT+tPA arm and 817 in the MT arm, were included in the meta-analysis. In each study, patient populations consisted of only tPA-eligible patients and all imaging and clinical outcomes were adjudicated by an independent and blinded core laboratory. Compared to MT alone, patients treated with MT+tPA had higher odds of eTICI ≥2b (OR = 1.34 [95% CI: 1.10; 1.63]). However, there were no statistically significant differences in the rates of 90-day mRS 0-2 (OR = 0.98 [95% CI: 0.77; 1.24]), 90-day mortality (OR = 0.94 [95% CI: 0.67; 1.32]), distal emboli (OR = 0.94 [95% CI: 0.25; 3.60]), or sICH (OR = 1.17 [95% CI: 0.80; 1.72]).

**Conclusions:** Administering tPA prior to MT may improve the rates of recanalization compared to MT alone in tPA-eligible patients being treated for AIS, but a corresponding improvement in functional and safety outcomes was not present in this review. Further studies looking at the role of tPA before mechanical thrombectomy in different cohorts of patients could better clarify the role of tPA in the treatment protocol for AIS.

## Introduction

Acute ischemic stroke (AIS) is caused by embolic or thromboembolic occlusion of a cervical or cerebral artery. Until recently, AIS treatment focused on intravenous thrombolysis with tissue plasminogen activator (IV-tPA), and eligible patients could be treated within 3–4.5 h of symptom onset (1). More recently, multiple randomized clinical trials demonstrated that mechanical thrombectomy (MT) results in superior functional outcomes compared to standard medical therapy, which includes IV-tPA treatment (2–9). Moreover, MT may be offered to eligible patients up to 24 h after symptom onset, which has expanded treatment options for thousands of AIS patients.

Currently, patients who are eligible for both MT and IV-tPA are recommended to receive both treatments (10). However, the effectiveness of MT has raised the question of whether IV-tPA offers any additional benefit in the treatment of AIS patients who are eligible for both therapies.

The recently reported DIRECT-MT, SKIP, DEVT, and MR CLEAN NO IV trials randomized patients to either MT alone to MT+tPA, and each of these trials failed to identify a significant difference in functional outcomes between these two treatment strategies (11–14). In addition, it is not clear whether MT+tPA results in a higher frequency of vessel recanalization compared to MT alone (11–14). We hypothesized that these individual studies were underpowered to detect significant differences in recanalization rates and functional outcomes between MT+tPA and MT alone patients. Therefore, we performed a systematic review and meta-analysis to consolidate the findings of all eligible randomized controlled trials that address this comparison.

Traditional reviews and meta-analyses require researchers to manually identify relevant literature across multiple databases, a process which can be inefficient and unorganized. The data extraction process too requires manually standardizing the format of data, units, and time point definitions, which lends itself to errors and can often be tedious (15). We sought to investigate a more streamlined approach, and thus performed this study using a novel semi-automated software platform (AutoLit, Nested Knowledge, Saint Paul, MN) that allows for the rapid identification, collation, synthesis, and analysis of data. Assessing the performance of this software platform was a secondary aim of this study.

## Methods

### Nested Knowledge Systemic Review Platform

**Figure 1:**
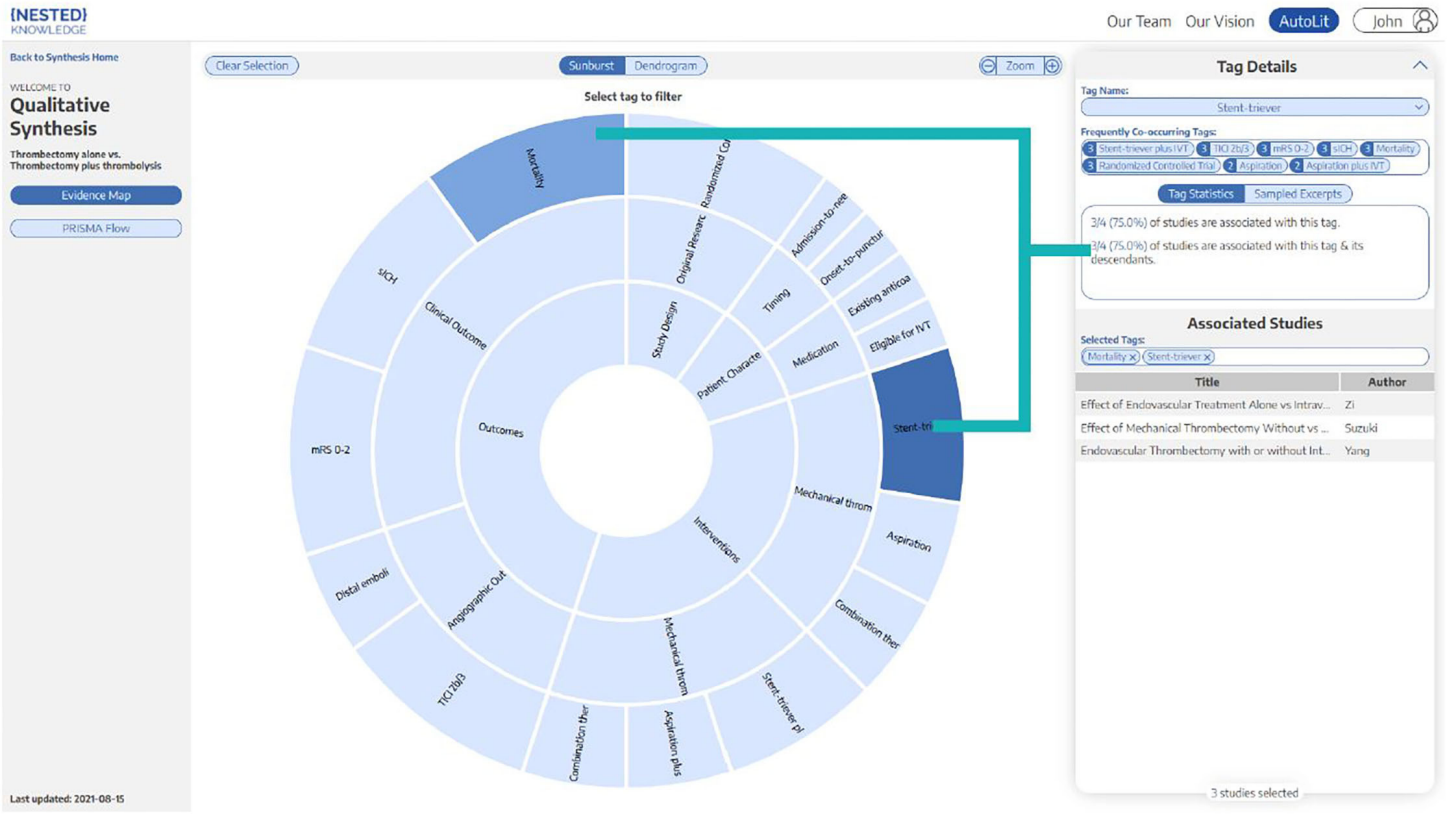
Patient demographics, interventions, and outcome variables. Sunburst diagram generated by Nested Knowledge depicting the variables and treatment arms of this study, with organization hierarchy from inside to outside. For example, “Clinical Outcomes” and “Angiographic Outcomes” are two sub-categories of “Outcomes”.

A PRISMA and MOOSE-compliant systematic review of the literature was undertaken on the PubMed database through the Nested Knowledge (NK) platform (Supplementary Video 1) Prior to study selection and screening, two authors (G.A. and J.J.H.) established the framework for the study by writing up a protocol for the systematic review that included acceptable study designs, intervention arms, patient characteristics to collect as baseline and outcome variables, as depicted in the NK sunburst diagram in Figure 1. These authors, also non-affiliates of NK, were also responsible for evaluating the functionality and efficiency of the NK platform as a secondary aim of this study.

### Literature Search and Study Selection

Initial search was completed on the PubMed database using the Application Program Interface (API) in the AutoLit platform; all study metadata and abstracts from the search results were also obtained *via* API. We included randomized control trials (RCTs) that reported a comparison of MT vs. MT+tPA in patients with anterior circulation AIS, and we captured these using the search strings:

1) “intravenous thrombolysis” AND (Trevo OR Solitaire)
2) [“without intravenous” AND (thrombectomy OR endovascular)] AND (clinical trial [Publication Type]).

Studies identified in the initial literature search were screened automatically by the AutoLit platform for two pre-configured automated exclusion criteria: (1) Published before 01/01/2010 and (2) Not published in English. Two independent raters (G.A. and K.H.) then used the AutoLit dual screening module to vet the remaining studies and included studies if they met the inclusion criteria: (1) RCTs of IV-tPA-eligible patients with treatment arms of patients treated with MT alone and patients treated with MT+tPA and (2) reported 90-day modified Rankin Scale score (mRS) 0–2, Expanded Thrombolysis in Cerebral Infarction (eTICI) ≥ 2b rate, 90-day mortality, distal emboli, and symptomatic intracranial hemorrhage (sICH) among the outcome variables. Studies that were not RCT by design or had IV-tPA-ineligible patients included in patient groups were excluded. Other manual exclusion criteria included:

- Includes pediatric patients
- Does not report an MT arm
- Does not report patient outcomes
- MERCI thrombectomy device used in MT arm.

After manual screening was completed, all disagreements between the two independent raters were independently adjudicated by another rater (N.H.). Detailed results of our study search, screening, and data extraction process are hosted on the Nested Knowledge website (https://nested-knowledge.com/).

### Extraction of Patient Characteristics and Outcome Variables

Extraction of the data from each selected study was completed by two authors (G.A. and K.H.) and confirmed for accuracy independently by two other authors (H.K. and D.M.H.). Patient characteristics collected included: age, sex, baseline National Institutes of Health Stroke Scale (NIHSS) score, onset-to-needle time, and onset-to-puncture time. Our primary outcome was rate of successful reperfusion, defined as eTICI ≥ 2b after MT. Secondary outcomes included functional independence (mRS score 0–2 at 90 days), 90-day mortality, sICH, and rate of distal emboli to new territories.

### Risk of Bias Assessment

The risk of bias and levels of evidence of each study was scored using the Scottish Intercollegiate Guidelines Network (SIGN) checklists for controlled clinical trials and cohort studies (16). Within their separate checklists, non-randomized controlled trials were rated no higher than 1+. The risk of bias assessment was completed independently by 2 authors (H.K. and D.M.H.). Any disagreements were discussed and resolved by a third author (J.M.P.).

### Statistical Analysis

Data was extracted within the Nested Knowledge interface, exported as a.csv file, and imported to RStudio (Version 1.3.959, RStudio, PBC, Boston, MA) running on R-4.0.2 for analysis. The “meta” (Version 4.18-0) and “metaphor” (Version 2.4-0) packages were used to perform meta-analyses (17, 18).

Effect sizes from each study were computed as logarithmically transformed odds ratios (ORs) with random-effects, Mantel-Haenszel weighting. Logarithmic transformations were used to correct for skewed marginal distributions and to shrink the influence of high leverage outliers. To aid in interpretation, logarithmically transformed pooled effect sizes were back-transformed to their original scale. The between-study variance component of random-effects models were estimated using restricted effects maximum likelihood (REML) with 95% CIs computed using the Q-profile method (19). Due to the small number of studies/patients included in the meta-analysis, 95% CIs around pooled effect sizes were calculated using Hartung-Knapp adjustment to provide a more conservative estimate of the true intervention effect and to reduce the risk of false positives (20). While significance tests of the pooled effect usually assume a standard normal distribution (typically using Wald-type tests), the Hartung-Knapp method is based on a *t*-distribution which typically produces larger CIs around the pooled effect size. 95% prediction intervals (PIs) around the pooled effect sizes were also calculated for each outcome measure using methods described by Higgins et al. (21).

## Results

### Nested Knowledge Platform Performance

We found the Nested Knowledge AutoLit platform to be expeditious, streamlined, and effective at sourcing the correct literature for our research question. No training was required to use this platform. Once appropriate search strings were identified, the process of running the search algorithm and excluding among 226 studies based on automated, pre-defined exclusion criteria took <1 min. The dual screening process for the remaining 185 studies took ~2 h in total per screener, with most of the time being allocated to verifying studies that were not clearly excludable by the abstract. Finally, data extraction for the four studies eventually included took <30 min. Most notably, the AutoLit platform maintained a full audit record of our search, screening criteria and activities, organized our interventions and data of interest, and provided an extraction environment with straightforward export for analysis (Supplementary Figure 1).

### Literature Search Results

Our initial search identified 221 studies, with 8 additional records identified through expert recommendation. After removing duplicates, a total of 226 articles were screened for inclusion. A total of 222 articles were excluded after screening based on title and abstract. A total of 3 full-text articles (DIRECT-MT, DEVT, SKIP) and 1 oral abstract presentation (MR CLEAN NO IV) were assessed for eligibility and included in the final quantitative meta-analysis (Supplementary Figure 2) (11–14). Among this study population, 817 patients were treated with MT alone, and 816 patients were treated with MT+tPA. The list of studies and patient characteristics are presented in Table 1, and study-specific patient outcomes are provided in Table 2.

**Table 1:** Patient characteristics of the four studies included in the meta-analysis.

	**Yang et al. (DIRECT-MT)**		**Zi et al. (DEVT)**		**Sukuzi et al. (SKIP)**		**Treurniet et al. (MR CLEAN NO IV)**	
**Treatment Arm**	**MT**	**MT±tPA**	**MT**	**MT±tPA**	**MT**	**MT±tPA**	**MT**	**MT±tPA**
**Number of patients**	327	329	116	118	101	103	273	266
**Age, years (median)**	69	69	70	70	74	76	N/A	N/A
**Sex**	189 M, 138 F	181 M, 148 F	66 M, 50 F	66 M, 52 F	56 M, 45 F	72 M, 31 F	N/A	N/A
**Baseline NIHSS (median)**	17	17	16	16	19	17	N/A	N/A

**Table 2:** Angiographic, functional, and safety outcomes of patients in the four studies included in the meta-analysis.

	**Yang et al. (DIRECT-MT)**		**Zi et al. (DEVT)**		**Sukuzi et al. (SKIP)**		**Treurniet et al. (MR CLEAN NO IV)**	
**Treatment Arm**	**MT**	**MT±tPA**	**MT**	**MT±tPA**	**MT**	**MT±tPA**	**MT**	**MT±tPA**
**eTICI** **≥** **2b**	243/306 (79.4%)	267/316 (84.5%)	100/116 (86.2%)	102/118 (86.4%)	91/101 (90.0%)	96/103 (93.2%)	214/273 (78.4%)	221/266 (83.1%)
**mRS 0–2**	119/327 (36.4%)	121/329 (36.8%)	63/116 (54.3%)	55/118 (46.6%)	60/101 (59.4%)	59/103 (57.3%)	134/273 (49.1%)	136/266 (51.1%)
**Mortality**	58/327 (17.7%)	62/329 (18.8%)	20/116 (17.2%)	21/118 (17.8%	8/101 (7.9%)	9/103 (8.7%)	56/273 (20.5%)	42/266 (15.8%)
**sICH**	14/327 (4.3%)	16/329 (4.9%)	7/115 (6.1%)	8/117 (6.8%)	6/101 (5.9%)	8/103 (7.8%)	16/273 (5.9%)	14/266 (5.3%)
**Distal Emboli**	35/327 (10.7%)	31/329 (9.4%)	19/113 (16.8%)	21/117 (17.9%)	N/A	N/A	N/A	N/A

### Risk of Bias and Qualitative Appraisal of Evidence

According to the SIGN checklists for controlled clinical trials, our risk of bias assessment identified 3 studies of high quality and 1 study of low quality. Of note, the MR CLEAN NO IV study was primarily considered low quality due to limited available information and the absence of peer-reviewed results at this time (12). The results of our quality appraisal are summarized in Supplementary Table I. Outcome reporting was fairly homogenous among the included studies, with all studies reporting eTICI ≥2b, sICH, 90-day mRS 0–2, and 90-day mortality. However, only two of the included studies reported rates of distal embolization to a new vascular territory. All included studies analyzed 90-day mRS as the primary endpoint of interest, with 2 studies dichotomizing mRS score as 0–2 (good clinical outcome) vs. 3–6 (poor clinical outcome) and the remaining 2 studies analyzing mRS score on its full ordinal scale. Qualitatively, two RCTs failed to demonstrate non-inferiority for MT alone compared to MT+tPA, whereas the other two RCTs suggested non-inferiority of MT alone compared to MT+tPA. Of note, none of the included studies demonstrated inferiority or superiority of either MT alone or MT+tPA.

### Successful Recanalization (eTICI ≥2b)

**Figure 2:**
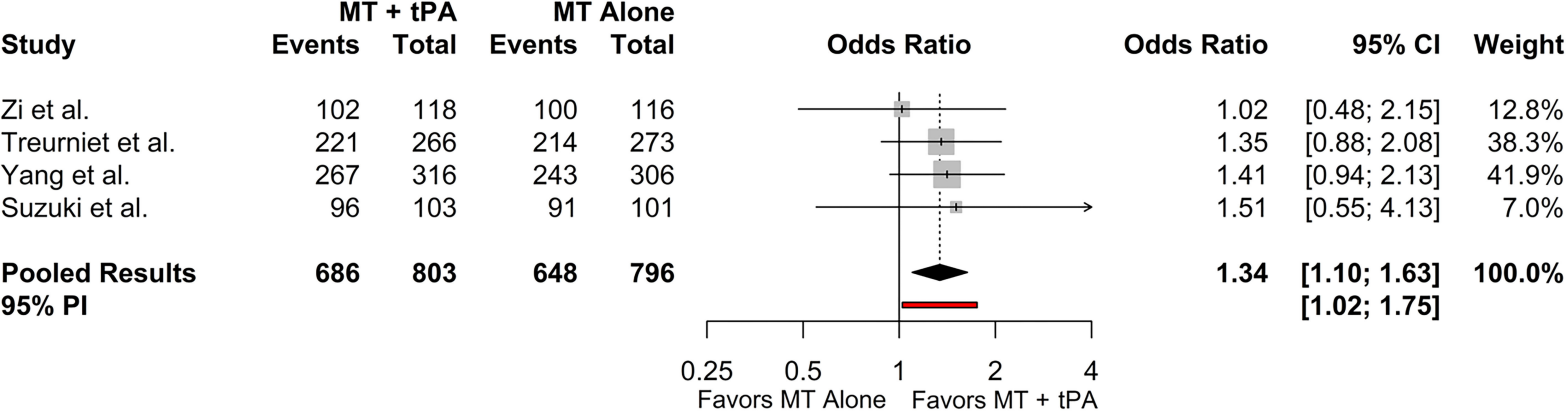
Forest plot of comparisons of eTICI ≥2b. Pooled results were computed *via* the Mantel-Haenszel method, using restricted effects maximum likelihood for estimation of the between-study variance component and Hartung-Knapp adjustment to calculate a 95% confidence interval (CI) around the pooled effect size. A 95% prediction interval (PI) for the pooled results are also displayed as a red bar.

All included studies reported successful recanalization defined by the eTICI ≥2b criteria and all results were adjudicated by an independent core laboratory blinded to treatment groups. The overall rate of eTICI ≥2b for the MT+tPA group was 85.6% (95% CI: 82.0–88.6%) vs. 83.0% (95% CI: 76.9–87.8%) in the MT alone group. The odds of achieving eTICI ≥2b were significantly higher in the MT+tPA group compared to the MT alone group (OR = 1.34 [95% CI: 1.10; 1.63]), *p* = 0.018; Figure 2). The estimated between-study heterogeneity unrelated to sampling error was low (*I*^2^ = 0.0% [95% CI: 0.0–27.3%]).

### Functional Independence (mRS 0–2 at 90 Days)

**Figure 3:**
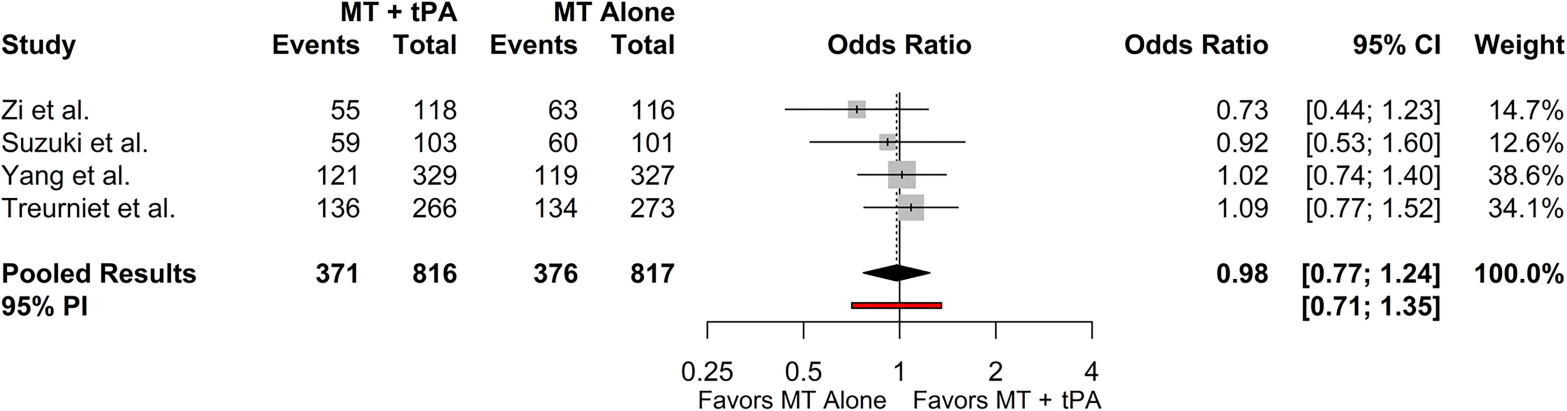
Forest plot of comparisons of mRS 0–2 at 90 days. Pooled results were computed *via* the Mantel-Haenszel method, using restricted effects maximum likelihood for estimation of the between-study variance component and Hartung-Knapp adjustment to calculate a 95% confidence interval (CI) around the pooled effect size. A 95% prediction interval (PI) for the pooled results are also displayed as a red bar.

The overall mRS 0–2 rate for the MT+tPA group was 47.4% (95% CI: 38.9–56.2%), and for the MT alone group was 49.2% (95% CI: 39.4–59.1%). There was no statistically significant difference in the odds of mRS 0–2 at 90 days between the MT+tPA group and the MT alone group (OR = 0.98 [95% CI: 0.77; 1.24], *p* = 0.787; Figure 3). The estimated between-study heterogeneity unrelated to sampling error ranged from low to high (*I*^2^ = 0.0% [95% CI: 0.0–72.4%]).

### Mortality at 90 Days

**Figure 4:**
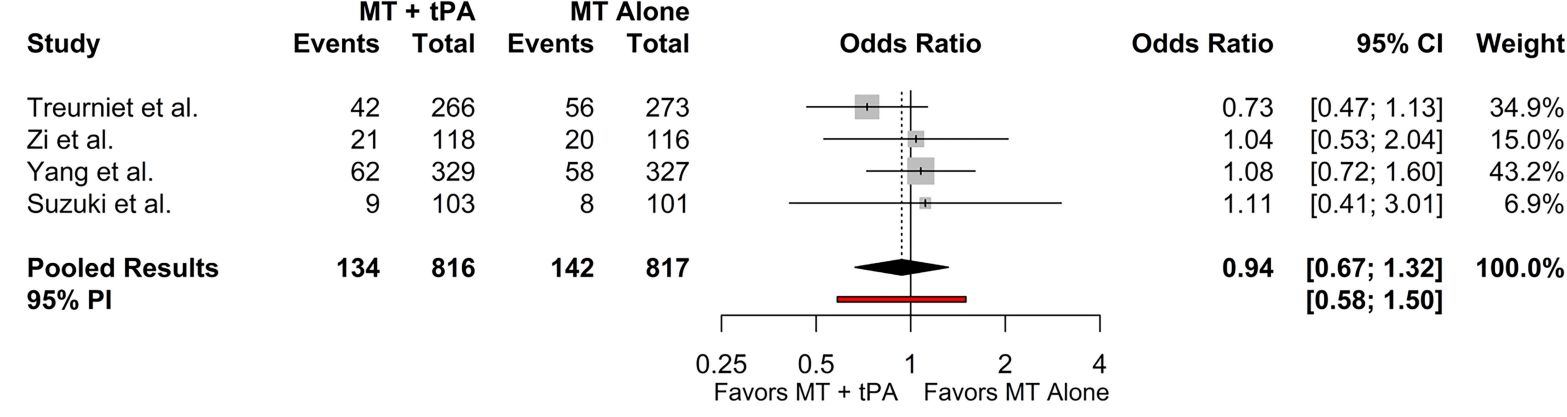
Forest plot of comparisons of mortality at 90 days. Pooled results were computed *via* the Mantel-Haenszel method, using restricted effects maximum likelihood for estimation of the between-study variance component and Hartung-Knapp adjustment to calculate a 95% confidence interval (CI) around the pooled effect size. A 95% prediction interval (PI) for the pooled results are also displayed as a red bar.

The overall mortality rate at 90 days for the MT+tPA group was 16.3% (95% CI: 13.2–19.9%), and for the MT alone group was 16.5% (95% CI: 12.3–21.8%). There was no statistically significant difference in the odds of mortality at 90 days between the MT+tPA group and the MT alone group (OR = 0.94 [95% CI: 0.67; 1.32], *p* = 0.582; Figure 4). The estimated between-study heterogeneity unrelated to sampling error ranged from low to high (*I*^2^ = 0.0% [95% CI: 0.0–76.5%]).

### Symptomatic Intracranial Hemorrhage (SICH)

**Figure 5:**
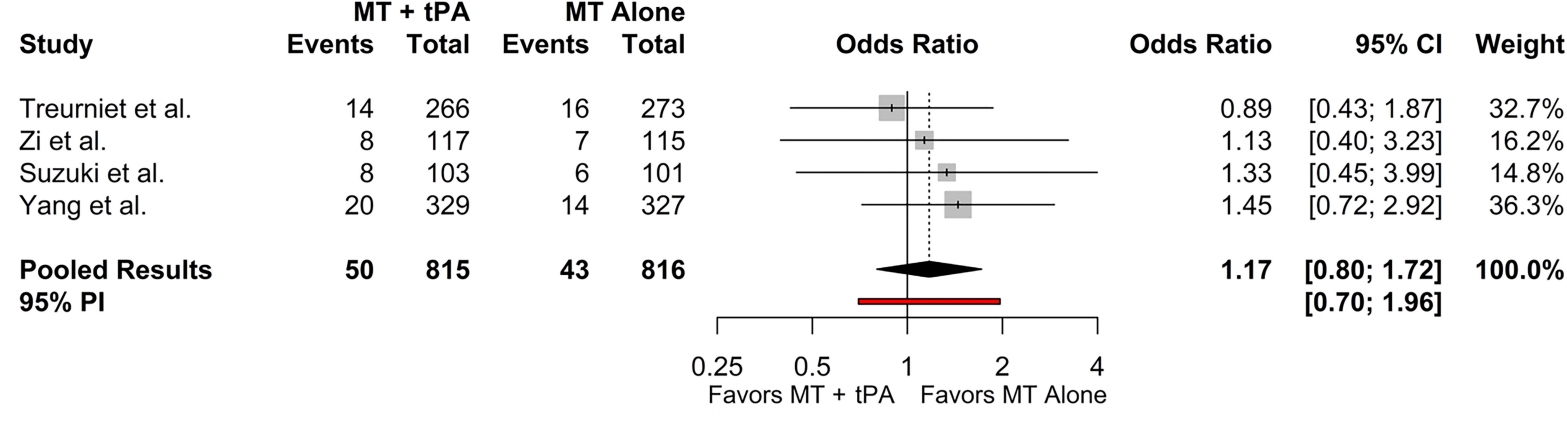
Forest plot of comparisons of sICH. Pooled results were computed *via* the Mantel-Haenszel method, using restricted effects maximum likelihood for estimation of the between-study variance component and Hartung-Knapp adjustment to calculate a 95% confidence interval (CI) around the pooled effect size. A 95% prediction interval (PI) for the pooled results are also displayed as a red bar.

Three studies reported sICH based on the Heidelberg Bleeding Classification criteria and one study reported sICH based on the SIT-MOST and NINDS criteria (22–24). Since the SKIP Randomized Clinical Trial reported sICH as both the SIT-MOST and NINDS criteria, we collected data based on the SIT-MOST criteria which more closely resembles the criteria defined by the Heidelberg Bleeding Classification, which was reported in the rest of the included studies. The overall rate of sICH for the MT+tPA group was 6.2% (95% CI: 4.7–8.1%), and for the MT alone group was 5.3% (95% CI: 4.0–7.1%). There was no statistically significant difference in the odds of sICH between the MT+tPA group and the MT alone group (OR = 1.17 [95% CI: 0.80; 1.72], *p* = 0.275; Figure 5). The estimated between-study heterogeneity unrelated to sampling error ranged from low to moderate (*I*^2^ = 0.0% [95% CI: 0.0–50.6%]).

### Distal Emboli to a New Territory Post-MT

**Figure 6:**
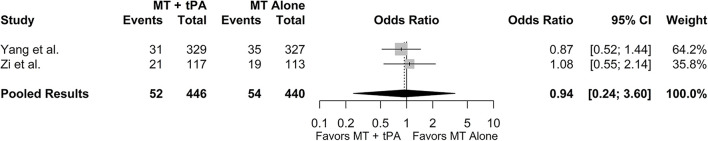
Forest plot of comparisons of distal embolization to new territory. Pooled results were computed *via* the Mantel-Haenszel method, using restricted effects maximum likelihood for estimation of the between-study variance component and Hartung-Knapp adjustment to calculate a 95% confidence interval (CI) around the pooled effect size.

The overall rate of distal emboli for the MT+tPA group was 12.9% (95% CI: 6.7–23.5%), and for the MT alone group was 13.1% (95% CI: 8.3–20.8%). There was no statistically significant difference in the odds of distal emboli between the MT+tPA group and the MT alone group (OR = 0.94 [95% CI: 0.25; 3.60], *p* = 0.659; Figure 6). While the point estimate of the *I*^2^ statistic indicates low heterogeneity (*I*^2^ = 0.0%), the true heterogeneity unrelated to sampling error is likely underestimated and this result should be interpreted with caution since only two studies were included in the analysis and a 95% CI could not be produced.

## Discussion

### Summary

In our meta-analysis of RCTs comparing outcomes of AIS patients treated with MT+tPA vs. MT alone, we found that patients treated with MT+tPA have higher odds of successful recanalization (eTICI ≥ 2b) (11–14) However, there were no detectable differences in rates of functional independence, 90-day mortality, sICH, or distal emboli between the MT+tPA and the MT alone groups. Overall, the results of this meta-analysis suggest that while angiographic outcomes after MT are superior with associated IV-tPA administration, it remains uncertain whether IV-tPA leads to improved functional outcomes or reduced complications.

### Takeaways: MT+IV-TPA vs. MT Alone

MT has transformed AIS treatment and results in improved clinical outcomes compared to medical therapy alone (2–9). Adequate revascularization of the cerebral circulation (eTICI >2b) after MT is important for the preservation of brain tissue, and the degree of revascularization has been correlated with improved clinical outcomes (25, 26). Whether adjunctive treatment with IV-tPA leads to higher revascularization rates and improved functional outcomes after MT remains uncertain. IV-tPA inherently increases the risk of complications such as intracranial hemorrhage, systemic hemorrhage, and angioedema, and the invasive nature of MT may further compound these risks (27). Combined treatment may be justified if patients have significantly better angiographic and functional outcomes, but reported RCTs (DIRECT-MT, DEVT, SKIP, and MR CLEAN-NO IV) have failed to identify significant differences between MT alone and MT+tPA treatment (11–14).

Our findings in this meta-analysis are consistent with a recent meta-analysis on this topic (28). However, of note, we replicated the findings of a manual study with a semi-automated approach through Nested Knowledge. We found that combined MT+tPA treatment increases the odds of achieving eTICI ≥ 2b reperfusion by a factor of 1.34. While superior reperfusion is expected to result in superior clinical outcomes, we did not detect any difference in functional outcomes, mortality, or sICH between MT and MT+tPA groups. The reasons for the discrepancy between angiographic and functional outcomes requires further study and is likely multifactorial (29–32). We did find a consistent trend toward favorable outcomes in each of these variables in MT+tPA patients, which suggests that a continued benefit for intravenous thrombolysis exists despite the strong effectiveness of MT. We also note that our analysis and future analyses would be bolstered by a more uniform reporting of near perfect (eTICI 2c) and perfect (TICI 3) revascularization, which are more strongly correlated with favorable outcomes (25, 26). It is possible that future RCTs and meta-analyses with larger patient numbers may clarify some of these findings.

### Nested Knowledge

In this study, we were able to test the process and functionality of the Nested Knowledge platform in performing efficient and focused systematic reviews. After a research protocol was written, search term selection for the automated scan of PubMed was intuitive and expeditious. Easy access to our inclusion and exclusion criteria, and a database of the reasons why each excluded study was removed was essential in preventing redundant work or a misguided review of literature. For each included study, the complete manuscript and recorded outcome variables were juxtaposed under the same function, which reduced the likelihood of error and served as a safety net to determine whether an included study truly fit our protocol. Finally, prior to formal statistical analyses, we utilized the Nested Knowledge automated data analysis feature to visually summarize potentially significant results that would warrant additional statistical analyses. We subjectively found the platform to be a streamlined, preferable alternative to standard practice in pooled research, and we appreciated the one-stop access to project details that it allowed.

### Limitations

The major limitation of this meta-analysis is the limited number of studies and the heterogeneity of techniques and devices classified as MT within data groups. Though this meta-analysis is strictly comprised of RCTs with well-balanced baseline patient characteristics, access to patient-level data would allow for a more robust analysis with rigorous control over differences in thrombectomy techniques and various other patient and study characteristics. This approach would also further clarify our research question. Furthermore, though we included four RCTs in this analysis, our study was relatively underpowered and could not detect small but clinically important differences in rates of our outcome variables between groups.

Another limitation is that the MR CLEAN-NO IV study results have not yet been published as a full-text, peer-reviewed article; as such, this study was classified as a low quality RCT associated with a relatively high risk of bias. Quality of data reporting also varied across studies, with only two studies reporting rates of distal embolization to new territory. Another minor limitation is that definitions of sICH varied, with 3 studies reporting sICH based on the Heidelberg Bleeding Classification and 1 study reporting sICH based on the SIT-MOST and NINDS criteria. Since the SKIP Randomized Clinical Trial reported sICH as both the SIT-MOST and NINDS criteria, we collected data based on the SIT-MOST criteria which more closely resembles the criteria defined by the Heidelberg Bleeding Classification which was reported in the rest of the included studies.

A final limitation is that the evaluation of the Nested Knowledge platform was subject to bias by the very nature of several authors being affiliated with the organization. However, we sought to mitigate this bias by having two authors (G.A. and J.J.H) who were not affiliated with NK evaluate the platform independently and report on their subjective experience.

## Conclusions

In this meta-analysis of the DIRECT-MT, DEVT, SKIP, and MR CLEAN-NO IV studies, we found superior revascularization after MT when IV-tPA was administered. However, these higher rates of revascularization in MT+tPA patients did not result in increased rates of functional independence or reduced complications compared to MT treatment alone. We also found the novel Nested Knowledge semi-automated systematic review platform to be an excellent and rapid tool for identifying and consolidating these studies.

## Data Availability Statement

The original contributions presented in the study are included in the article/Supplementary Material, further inquiries can be directed to the corresponding author/s.

## Author Contributions

All contributing authors of this work have: 1. Made a substantial contribution to the concept and design, acquisition of data or analysis and interpretation of data. 2. Drafted the article or revised it critically for important intellectual content. 3. Approved the version to be published.

## Funding

This research was supported in part by a generous grant from the Hawai'i Community Foundation to JH.

## Conflict of Interest

JP works for and holds equity in Nested Knowledge, Inc., and Superior Medical Experts, Inc. KH works for Nested Knowledge, Inc. KK works for and holds equity in Nested Knowledge, Inc., works for Conway Medical LLC, and holds equity in Superior Medical Experts, Inc. NH works for and holds equity in Nested Knowledge, Inc. JH is a consultant for Medtronic and MicroVention and is a member of the scientific and medical advisory board for iSchemaView. The remaining authors declare that the research was conducted in the absence of any commercial or financial relationships that could be construed as a potential conflict of interest.

## Publisher's Note

All claims expressed in this article are solely those of the authors and do not necessarily represent those of their affiliated organizations, or those of the publisher, the editors and the reviewers. Any product that may be evaluated in this article, or claim that may be made by its manufacturer, is not guaranteed or endorsed by the publisher.

## Supplementary Material

Supplementary Figure 1 Step-by-step schematic of the systematic review process in Nested Knowledge. The first step is conceiving a study protocol and a nest with arms, baseline variables, and outcomes to guide the rest of the process. This is followed by an semi-automated literature search based on search strings, screening of studies for inclusion, tagging of included studies with relevant labels from the nest, extraction of data, and cursory review of the automated data synthesis prior to a full analysis. Each of these steps is sequentially included in this figure.

Supplementary Figure 2 PRISMA diagram of search records and included studies automatically generated by the AutoLit platform.

Supplementary Table 1 Risk of bias assessment per the Scottish Intercollegiate Guideines Network for the four studies included in this meta-analysis.

Supplementary Video 1 Video walkthrough of the steps in the systematic review process on the Nested Knowledge living platform and other functionality features.
