# Supplementary figures and images for: Mechanical Thrombectomy With and Without Intravenous Tissue Plasminogen Activator for Acute Ischemic Stroke: A Systematic Review and Meta-Analysis Using Nested Knowledge
Source: Front Neurol. 2021 Dec 17;12:759759. doi: 10.3389/fneur.2021.759759 (PMC8719452; doi:10.3389/fneur.2021.759759)

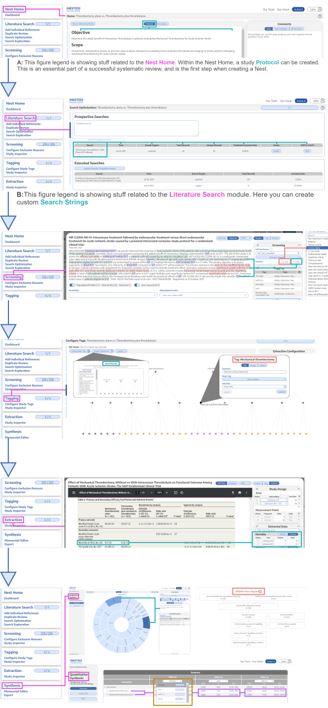

Supplement: Supplementary Figure 1 — Step-by-step schematic of the systematic review process in Nested Knowledge. The first step is conceiving a study protocol and a nest with arms, baseline variables, and outcomes to guide the rest of the process. This is followed by an semi-automated literature search based on search strings, screening of studies for inclusion, tagging of included studies with relevant labels from the nest, extraction of data, and cursory review of the automated data synthesis prior to a full analysis. Each of these steps is sequentially included in this figure. [file Image_1.jpeg]

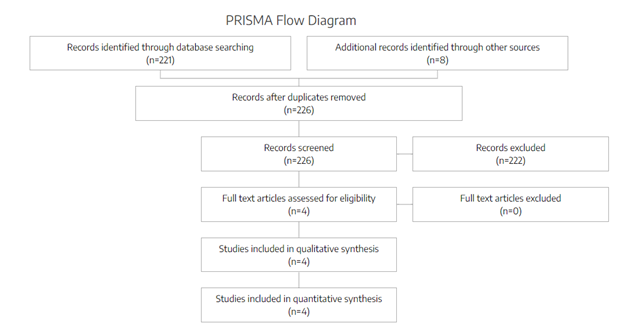

Supplement: Supplementary Figure 2 — PRISMA diagram of search records and included studies automatically generated by the AutoLit platform. [file Image_2.tif]
